# Native contrast visualization and tissue characterization of myocardial radiofrequency ablation and acetic acid chemoablation lesions at 0.55 T

**DOI:** 10.1186/s12968-020-00693-1

**Published:** 2021-05-06

**Authors:** Aravindan Kolandaivelu, Chris G. Bruce, Rajiv Ramasawmy, Dursun Korel Yildirim, Kendall J. O’Brien, William H. Schenke, Toby Rogers, Adrienne E. Campbell-Washburn, Robert J. Lederman, Daniel A. Herzka

**Affiliations:** 1 Cardiovascular Branch, Division of Intramural Research, National Heart Lung and Blood Institute, National Institutes of Health, Bethesda, MD 20892 USA; 2 Division of Cardiology, Department of Medicine, Johns Hopkins University School of Medicine, Baltimore, MD USA; 3 Biophysics and Biochemistry Branch, Division of Intramural Research, National Heart Lung and Blood Institute, National Institutes of Health, Bethesda, MD USA; 4 Institute of Biomedical Engineering, Bogazici University, Istanbul, Turkey; 5 Medstar Washington Hospital Center, Washington, DC USA

**Keywords:** Heart, Catheter ablation, Image-guided intervention, Tissue characterization, RF ablation, Chemoablation, Arrhythmias, Myocardial ablation

## Abstract

**Purpose:**

Low-field (0.55 T) high-performance cardiovascular magnetic resonance (CMR) is an attractive platform for CMR-guided intervention as device heating is reduced around 7.5-fold compared to 1.5 T. This work determines the feasibility of visualizing cardiac radiofrequency (RF) ablation lesions at low field CMR and explores a novel alternative method for targeted tissue destruction: acetic acid chemoablation.

**Methods:**

N = 10 swine underwent X-ray fluoroscopy-guided RF ablation (6–7 lesions) and acetic acid chemoablation (2–3 lesions) of the left ventricle. Animals were imaged at 0.55 T with native contrast 3D-navigator gated T1-weighted T1w) CMR for lesion visualization, gated single-shot imaging to determine potential for real-time visualization of lesion formation, and T1 mapping to measure change in T1 in response to ablation. Seven animals were euthanized on ablation day and hearts imaged ex vivo. The remaining animals were imaged again in vivo at 21 days post ablation to observe lesion evolution.

**Results:**

Chemoablation lesions could be visualized and displayed much higher contrast than necrotic RF ablation lesions with T1w imaging. On the day of ablation, in vivo myocardial T1 dropped by 19 ± 7% in RF ablation lesion cores, and by 40 ± 7% in chemoablation lesion cores (p < 4e−5). In high resolution ex vivo imaging, with reduced partial volume effects, lesion core T1 dropped by 18 ± 3% and 42 ± 6% for RF and chemoablation, respectively. Mean, median, and peak lesion signal-to-noise ratio (SNR) were all at least 75% higher with chemoablation. Lesion core to myocardium contrast-to-noise (CNR) was 3.8 × higher for chemoablation. Correlation between in vivo and ex vivo CMR and histology indicated that the periphery of RF ablation lesions do not exhibit changes in T1 while the entire extent of chemoablation exhibits T1 changes. Correlation of T1w enhancing lesion volumes indicated in vivo estimates of lesion volume are accurate for chemoablation but underestimate extent of necrosis for RF ablation.

**Conclusion:**

The visualization of coagulation necrosis from cardiac ablation is feasible using low-field high-performance CMR. Chemoablation produced a more pronounced change in lesion T1 than RF ablation, increasing SNR and CNR and thereby making it easier to visualize in both 3D navigator-gated and real-time CMR and more suitable for low-field imaging.

**Supplementary Information:**

The online version contains supplementary material available at 10.1186/s12968-020-00693-1.

## Introduction

Catheter ablation is increasingly used to treat ventricular tachycardia (VT) in structural heart disease, but arrhythmia commonly recurs after ablation [1–3]. Procedure failure is likely due to incomplete ablation of arrhythmogenic tissue. Cardiovascular magnetic resonance (CMR) is of increasing interest for characterizing the arrhythmogenic substrate [4–6], and for visualizing the location and extent of ablated tissue with higher contrast and resolution compared to imaging modalities like computed tomography, ultrasound, and single photon emission computed tomography (SPECT)/positron emission tomography (PET) [7–10]. However, performing cardiac ablation procedures under CMR guidance has been limited by the ability to distinguish permanent from transient tissue injury acutely after ablation [11], and the concern for CMR scanning induced heating of catheters and guidewires [12–16].

Accurate determination of radiofrequency (RF) ablation induced tissue necrosis is key for intra- or post-procedural lesion assessment. Gadolinium-based contrast agents have been applied but are not preferred as lesion hyperenhancement overestimates the extent of necrosis until days or weeks after ablation when transient tissue injury and edema have resolved [11, 17–19]. In addition, contrast dose limits restrict the ability to perform repeated lesion assessment during a procedure. Recently, techniques using the native T1 contrast created at the core of RF ablation lesions have demonstrated excellent correlation with extent of necrosis, without need to administer an exogenous contrast agent [17, 20, 21]. As we demonstrate in this work, assessment of lesion necrosis resulting from RF ablation using native T1 contrast is also feasible at low field.

RF ablation induces tissue necrosis by a thin rim of resistive heating adjacent to the catheter electrode [22]. This limits ablation of VT resulting from arrhythmogenic substrate deeper within the myocardium [5], and appears to restrict ablation through arrhythmogenic tissue like scar and fat which have thermally insulating properties [20]. As an alternative, chemoablation by transcatheter injection of caustic agents such as ethanol or acetic acid can generate focal tissue necrosis deeper within the myocardium [23]. As we demonstrate, chemoablation with acetic acid can also generate the desired T1 contrast that facilitates assessment of necrotic RF ablation lesion location and extent.

Performing ablation procedures under real-time CMR guidance could allow CMR lesion assessment to be smoothly integrated into procedure workflow [24]. The concern for RF heating of interventional devices has also slowed clinical translation of cardiac ablation procedures with intra-procedural CMR guidance. As the specific absorption rate (SAR) is proportional is to the square of field strength (B_0_), low field (0.55 T) imaging reduces heating around sevenfold relative to 1.5 T and 30-fold relative to 3 T [25]. With lower field strength, additional existing devices can be safely deployed during intervention without risk of heating and design and fabrication of new devices, e.g. chemoablation needles, is simplified [25–27].

Reduced field strength directly impacts signal-to-noise ratio (SNR) and achievable image resolution. Three-dimensional (3D) imaging techniques and decreased acquisition bandwidth can both recover SNR yet do so at the expense of scan duration. Hence, this work explores the feasibility of RF ablation lesion visualization at lower field strength using 3D as well as single-shot 2D T1-weighted (T1w) imaging. In addition, acetic acid chemoablation by direct myocardial injection is explored as a potential alternative for CMR-guided ablation.

## Methods

### Animal preparation

Animal protocols were reviewed and approved by the Animal Care and Use Committee at the National Institutes of Health. Cardiac ablation was performed in Yorkshire swine (N = 10). Animals were studied under general anesthesia with isoflurane (1–2%) and mechanical ventilation. Trans-septal puncture was performed using femoral venous access with fluoroscopic and intra-cardiac echocardiography (ICE) guidance (Acuson SC2000, Siemens Healthineers, Erlangen, Germany). A pacing catheter was placed in the right ventricle (RV) for overdrive pacing during RF ablation to suppress ablation-induced ventricular fibrillation (VF). A steerable sheath (Agilis, Abbott Inc. Abbott Park, Illinois, USA) was advanced trans-septally across the mitral valve. Ablation catheters were advanced into the left ventricle (LV) through the steerable sheath and tip location and tissue contact confirmed by fluoroscopy and ICE.

RF ablation was performed first using a 3.5 mm open irrigated tip catheter (Thermocool, Biosense Webster, Irvine, California, USA). Power delivery was 40–50 W for 30 s to one minute per location. Contact force sensing was not available on the RF ablation system available for this study, but ablation was performed in locations where catheter deflection could be directed into the tissue. Deflection was maintained during ablation in a way likely to generate at least 15 g contact force and manually adjusted targeting an impedance drop of 15–20 Ω. Six to seven RF ablations were performed per animal. Defibrillation was performed as needed for VF. To suppress VF, 150 mg of intravenous amiodarone was administered prior to ablation and RV overdrive pacing at around 150 bpm was initiated prior to each ablation. RV pacing was stopped ~ 30 s after each ablation for blood pressure recovery.

Chemoablation was performed using either a prototype injection catheter or a commercially available catheter with extendable needle-like tip (Wingman, Reflow Medical, San Clemente, California, USA) delivered through a steerable sheath (Agilis, Abbott Inc.) and co-axial 8 Fr multi-purpose (MPX) guide catheter. After deploying the needle-like tip up to 5 mm into the sub-endocardium under ICE, slow manual injection of 1 ml of either 50% or 100% glacial acetic acid (Avantor, Macron Fine Chemicals, VWR International Holdings, Radnor, Pennsylvania, USA) was performed over a period of one to two minutes. Two to three chemo-ablation injections were performed per animal. Care was taken to avoid sites of previous RF or chemoablation lesions to facilitate lesion segmentation. Chemoablation did not require overdrive pacing for VF suppression.

All animals were imaged within two hours of the first RF ablation and within 30 min of the generation of the last chemoablation lesion. This delay was felt acceptable based on previously observed stability of native-T1 ablation lesion enhancement from within 10 min to a week after RF ablation [20]. N = 7 animals were euthanized after acute post-ablation CMR. To observe changes in lesions through the healing process, 3 animals were reimaged 21 days post ablation and euthanized after their chronic post-ablation CMR. Hearts were excised and underwent ex vivo CMR. One acute animal died immediately after imaging and no ex vivo* C*MR was possible.

### In Vivo CMR

Imaging was carried out on a 0.55 T CMR scanner (prototype MAGNETOM Aera, Siemens Healthineers, Erlangen, Germany) using the standard spine coil array and either a six-element body array or a custom 16-channel array (Quality Electrodynamics, Mayfield, Ohio, USA).

Three dimensional respiratory-navigator gated T2-weighted (T2w) imaging was used to highlight edema surrounding lesions. A T2-prepared [28], whole-heart acquisition with a balanced steady-state free precession (bSSFP) readout was acquired during ventricular diastasis. 3D T1w inversion recovery (IR) imaging with a long inversion time (TWILITE) was carried out as described in [20] and [17]. Briefly, using 2-RR triggering, a non-selective IR pulse was applied followed by a long inversion time (TI ~ 550–600 ms) to suppress the signal from the blood pool and increase contrast between normal myocardium and ablation lesions. A respiratory-navigator-gated bSSFP sequence was used for image acquisition. To determine the changes in underlying tissue T1 relaxation rate after ablation, 2D breath-hold T1 mapping was carried out with a modified version of saturation recovery single-shot acquisition (SASHA) [29]. For the three animals reimaged at 21 days post ablation, 3D respiratory navigator-gated early and late delayed gadolinium enhancement (EGE, LGE) were additionally acquired after 2 × dose (0.2 ml/kg) of gadolinium-based contrast agent (GBCA) (Gadavist, Bayer Healthcare, Berlin, Germany) on both Day 0 and Day 21.

Tables 1 and 2 summarize typical image acquisition parameters used in this work. TI for in vivo T1w scans and *k*-lines per heartbeat were adjusted based on heart rate resulting in varying acquisition times. Gadolinium contrast agent was only used for animals in the Day 21 cohort during both imaging sessions. Nominal acquisition time does not include respiratory navigator efficiency which varied between 40 and 65%.

**Table 1:** Typical CMR imaging parameters for in vivo scans

In vivo scan	Respiratory motion compensation	Imaging technique	Acquired / reconstructed spatial resolution (mm^3)^	TR / TE (ms)	Flip angle (deg)	Receiver bandwidth (Hz/pixel)	Nominal acquisition time, range (min / heartbeats)	K-lines per heartbeat (min–max)	Acceleration factor (GRAPPA)	Other parameters
*Pre Gd-contrast*										
IR T1w 3D 2RR	Navigator	Segmented bSSFP	1.0 × 1.0 × 3.0–0.9 × 0.9 × 3.0/1.0 × 1.0 × 1.5–0.9 × 0.9 × 1.5	4.80 / 2.40	90	496	23.8–31.6 / 2 × 715–950	12–16	None	2RR gating, TI = 550–600 ms
T2w 3D	Navigator	Segmented bSSFP	0.9 × 0.9 × 3.0 /0.9 × 0.9 × 1.5	4.40 / 2.20	90	676	14.0–18.7 / 840–1120	12–16	None	T2Prep TE = 45 ms
T1 Mapping SAX Stack	Breath-hold	SASHA	1.63 × 1.63 × 8	3.15 / 1.28	80	543	0.28 / 17	72	2	17 Images/map, 8 × Tsat = 104 ms; 4xTsat = 250 ms 4 × Tsat = 350 ms 7/8 partial Fourier
Single Shot IR T1w 2D 2RR	Free-breathing	Segmented bSSFP	2.51 × 1.88 × 6 / 0.94 × 0.94 × 6	4.91 / 1.92	90	250	2 beats / 2	53	2	TI = 500–600 ms Assymetric readout
*Post Gd-contrast*										
EGE 3D	Navigator	Segmented bSSFP	1.5 × 1.5 × 6 /1.5 × 1.5 × 3	4.22 / 2.11	90	501	5.6–7.5 / 336–450	12–16	None	TI: 250 ms
LGE 3D	Navigator	Segmented bSSFP	0.9 × 0.9 × 3.0 /0.9 × 0.9 × 1.5	4.80 / 2.40	90	496	16.8–22.4 / 1010–1346	12–16	None	TI: 300 ms

*T1w* T1-weighted, *T2w* T2-weighted, *EGE* early gadolinium enhancement, *LGE* late gadolinium enhancement, *bSSFP* balanced steady state free precession, *SPGR* RF-spoiled gradient echo; *SASHA* saturation recovery single-shot acquisition, *DESPOT* Driven equilibrium single pulse observation of T1, *GRAPPA* generalized autocalibrating partially parallel imaging; *TI*  Inversion time, *T*_*SAT*_ Saturation delay time, *Gd* Gadolinium

**Table 2:** Typical CMR imaging parameters for ex vivo scans

Ex vivo scan	Imaging technique	Acquired / reconstructed spatial resolution (mm^3)^	TR / TE (ms)	Flip angle (deg)	Receiver bandwidth (Hz/pixel)	Acquisition time (h)	Acceleration factor (GRAPPA)	Other parameters
T1-W 3D -Mid Res	SPGR	0.5 isotropic / 0.5 isotropic	25.0 / 5.23	40	130	~ 2	None	6 Averages
T1-W 3D -High Res	SPGR	0.25 × 0.25 × 0.5 / 0. 25 Isotropic	25.0 / 5.23	40	130	10–12	None	24–30 Averages
DESPOT T1 Mapping	SPGR	1.0 isotropic / 1.0 isotropic	30 / 4.72	11°–56°	130	1.35	None	10 Flip angles 5°steps

*SPGR* RF-spoiled gradient echo, *DESPOT* drive equilibrium single pulse observation of T1, *GRAPPA* generalized autocalibrating partially parallel imaging

### Ex vivo CMR

Day 0 acute animals were imaged without contrast. Day 21 chronic animals were administered an additional 2 × dose of gadolinium 20 min before being euthanized. To achieve a diastolic conformation for imaging, the heart was excised and filled with either a liquid epoxy (Task 5, Smooth-On, Inc., Easton, Pennsylvania, USA) or a silicon rubber (MoldStar 31 T, Smooth-On, Inc.). A plastic screw was inserted into the aorta before curing and later used to secure the heart for imaging while submerged in perfluorocarbon (Fomblin, Solvay). 3D T1 mapping using driven equilibrium single pulse observation of T1 (DESPOT) [30], and 3D T1w images were acquired. Due to residual coherences in DESPOT T1 mapping, a correction method was determined using the T1MES phantom [31], as described in the Additional file 1.

### Histology

Four hearts (3 Day 0 acute) were explanted and preserved for histological analysis in 4% buffered formaldehyde (Formalin, Sigma Aldrich, St. Louis, Missouri, USA). Hearts were sliced at 3–5 mm slice thickness. After paraffin embedding tissues were stained with H&E and Masson's Trichrome.

### Data analysis

All lesions from in vivo and ex vivo CMR including T1 mapping, T1w imaging and LGE, were contoured in Osirix (Pixmeo, Geneva, Switzerland). Contours were exported and processed in MATLAB (Mathworks, Natick, Massachusetts, USA). SNR was measured using the mean signal value of the 3D ROIs divided by the standard deviation of a background noise region of interest (ROI). The first case in the study was not included in this analysis due to distinctly different prescriptions. Contrast-to-noise ratios (CNRs) were obtained by direct subtraction of SNRs. CNR was calculated between normal myocardium, blood pool, necrotic lesion core, and lesion external periphery (the area immediately surrounding the lesion ROI). For T1 mapping results, changes in lesion T1 relative to normal myocardial T1 was determined.

For accurate lesion volume analysis, confluent lesions were excluded as signal intensities could change if lesion core and lesion periphery regions of different lesions overlapped. For the determination of lesion volume, enhancement in T1w imaging was determined by $SI\ge {\mu }_{Myo}+{2\sigma }_{Myo}$, where SI is the signal intensity, ${\mu }_{Myo}$ is the local mean myocardial signal, and ${\sigma }_{Myo}$ is the standard deviation of the myocardial signal. Conversely, the total lesion volume was calculated by $SI\ge {\mu }_{Myo}-{2\sigma }_{Myo}$, which excluded pixels within the manually drawn ROI that were hypointense and represent coagulum at the lesion core or in needle tracks.

## Results

High-resolution 3D lesion assessment proved feasible using low field CMR. In cases where RF ablation lesions overlapped or were confluent, the group of lesions were treated as a single ablation. In total, 50 RF ablation lesions were visualized in vivo as were 23 chemoablation lesions. Of those, 37 (74%) RF ablation and 18 (78%) chemoablation lesions were in the acute Day 0 cohort, and of those 13 (35%) and 15 (78%) were non-overlapping with any other lesion.

**Fig. 1:**
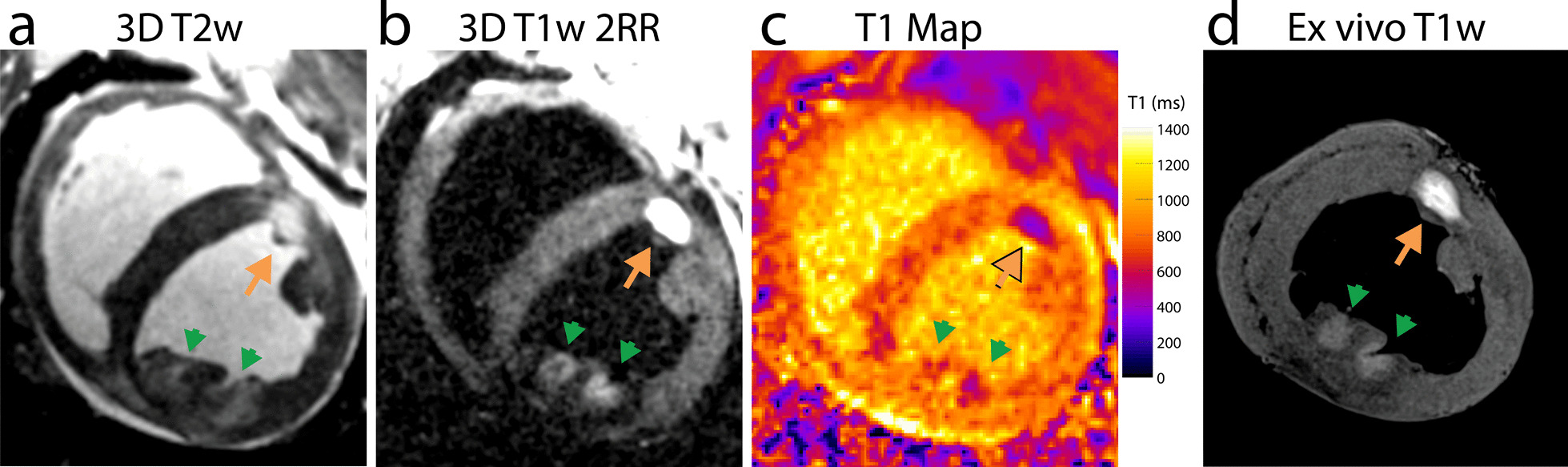
Native contrast cardiovascular magnetic resonance (CMR) of radiofrequency (RF) ablation (green arrows) and chemoablation (orange arrows) in the left ventricle on the day of ablation. **a** The boundary of T2 weighted (T2w) enhancement (edema) at sites of ablation is more diffuse than the more discrete lesion borders visualized by (**b**) T1 weighted (T1w) imaging. **c** T1 mapping shows a lower T1 for chemoablation lesion cores compared to RF ablation, corresponding to the greater degree of enhancement noted on T1w imaging. **d** Ex vivo native contrast T1w imaging showed the same lesion extent and relative enhancement as in vivo T1w imaging. Overall, both RF and chemoablation lesions were well visualized on the day of ablation

Figure 1 shows LV short axis images acquired on the day of ablation with both RF ablation and chemoablation lesions. T2 weighter (T2w) imaging (Fig. 1a) displays signal enhancement with lack of distinct borders likely due to diffuse edema at the lesion periphery. T2w enhancement is also seen in the lesion core, though the degree of enhancement varies with ablation type. Native contrast T1w imaging (Fig. 1b) shows signal enhancement that is confined to the lesion core for both RF ablation and chemoablation. Hypoenhancement surrounding T1w lesions correlates well with location of edema in Fig. 1a. Chemoablation displays higher contrast between lesion core and myocardium, indicating a larger drop in T1. T1 mapping (Fig. 1c) with SASHA confirms the changes in T1 after ablation and quantifies a larger decrease with chemoablation from 788 ± 62 ms in normal myocardium to 562 ± 31 ms (− 28%) in RF ablation and 458 ± 56 ms (− 42%) in chemoablation for this image. Comparison with higher-resolution ex vivo T1w imaging (Fig. 1d) confirms these findings including the extent of lesions. A second example highlighting the correlation of T2w imaging with the hypointense rim surrounding the RF ablation lesion can be seen in Additional file 2: Figure S1. Due to the relatively long scan times, it is probable that lesion morphology evolved between the acquisition of the T2w images (acquired first) and the T1w images (acquired after) as edema surrounding the lesion continued to increase in extent and intensity [24]. Despite this expected temporal evolution, there was excellent correspondence between hyperintensity on T2w imaging, and hypointensity on T1w imaging.

**Fig. 2:**
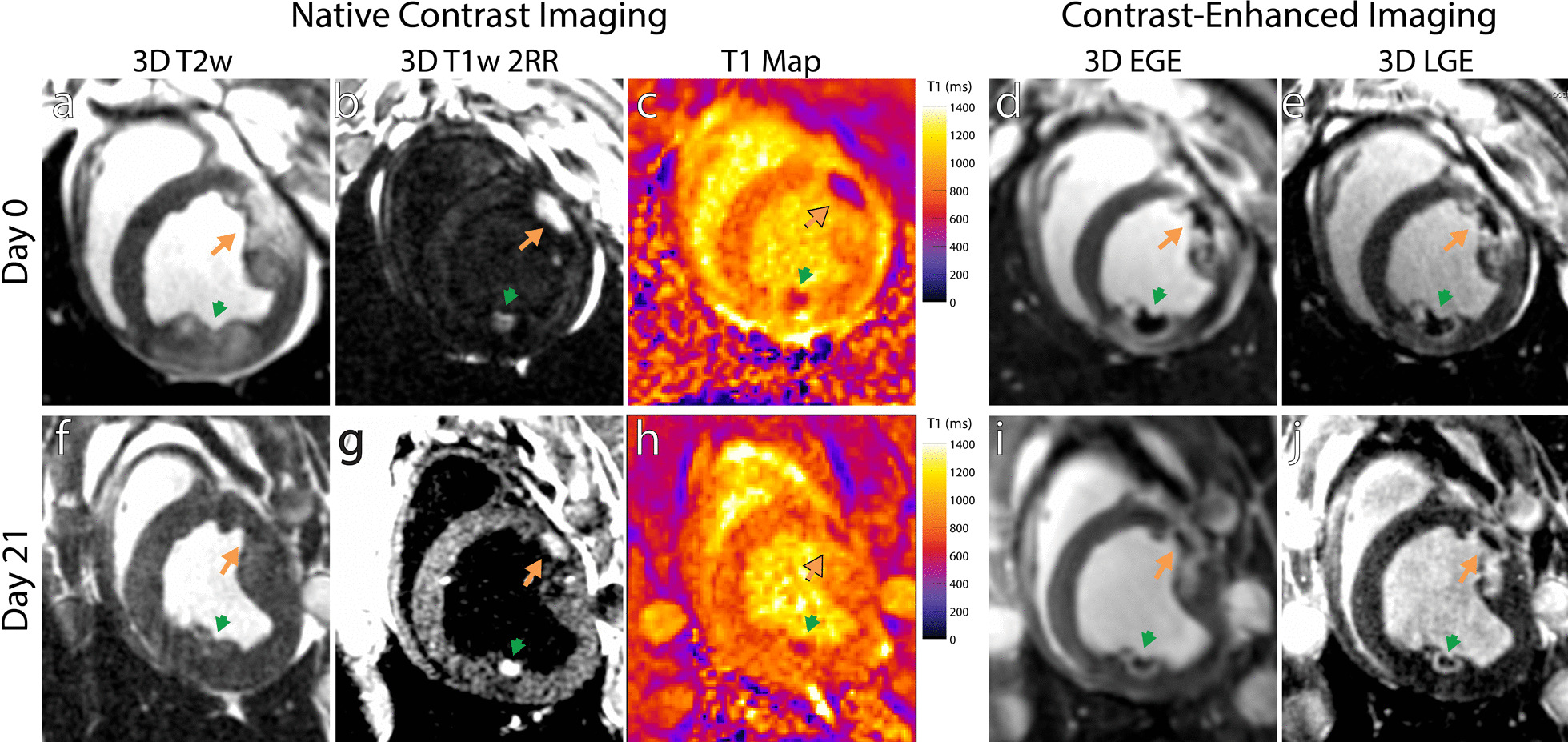
Time course of ablation lesion appearance from acutely after ablation (Day 0, top row) to Day 21 after ablation (bottom row) for native contrast imaging (three leftmost columns) and gadolinium-based contrast enhanced imaging (two rightmost columns) of RF ablation lesions (green arrows) and chemoablation lesions (orange arrows). **a**, **f** Edema, as imaged by T2w enhancement, around areas of ablation on Day 0 is no longer seen by Day 21. **b**, **g** T1w enhancement seen on Day 0 can still be seen on Day 21. **c**, **h** However, T1 maps show a reduced magnitude of T1 decrease on day 21 compared to day 1. Early gadolinium enhanced (EGE) imaging (**d**, **i**) and late-gadolinium enhanced (LGE) imaging (**e**, **g**) show a hypo-enhancing lesion core and an enhancing lesion periphery. The peripheral area of enhancement diminished by Day 21 compared to Day 0, corresponding to reduction of edema by T2w imaging. Both RF ablation and chemoablation demonstrate scar observed on Day 21 post ablation

Figure 2 illustrates changes in CMR lesion appearance from acutely after ablation (Day 0) to chronically after ablation (Day 21). Diffuse T2w enhancement seen acutely after ablation is no longer present chronically, likely corresponding to resolution of tissue edema. As in Fig. 1, native contrast T1w imaging acutely after ablation shows greater enhancement for chemoablation compared to RF ablation, in correspondence with a greater decrease in T1 measured by mapping. However, by Day 21, T1w imaging shows a reduction in the size of enhancing ablation lesions and hypoenhancement of the lesion periphery. Prior work evaluating RF ablation with T1w imaging showed this change corresponded to replacement of acutely enhancing necrotic tissue with chronic hypoenhancing fibrotic tissue [17]. Acute and chronic gadolinium-enhanced lesion appearance is also illustrated. At Day 0, EGE and LGE imaging displays signal voids at lesion cores, typically associated microvascular obstruction or coagulum after acute myocardial infarction. Previous work on RF ablation has demonstrated good correlation between this area of hypointensity and necrotic tissue [17, 32]. However the hypoenhancing region shrinks over time after contrast injection, making it an ambiguous measure of necrotic lesion extent [7, 17, 20]. Acutely after ablation, the lesion boundary in contrast enhanced imaging overestimates the extent of necrosis due to edematous tissues accumulating contrast media [17, 19, 32]. By Day 21, the area of hyperenhancement in contrast enhanced imaging accurately reflects the extent of the lesion as edema has dissipated and the necrotic core is surrounded by a newly deposited fibrous boundary.

**Fig. 3:**
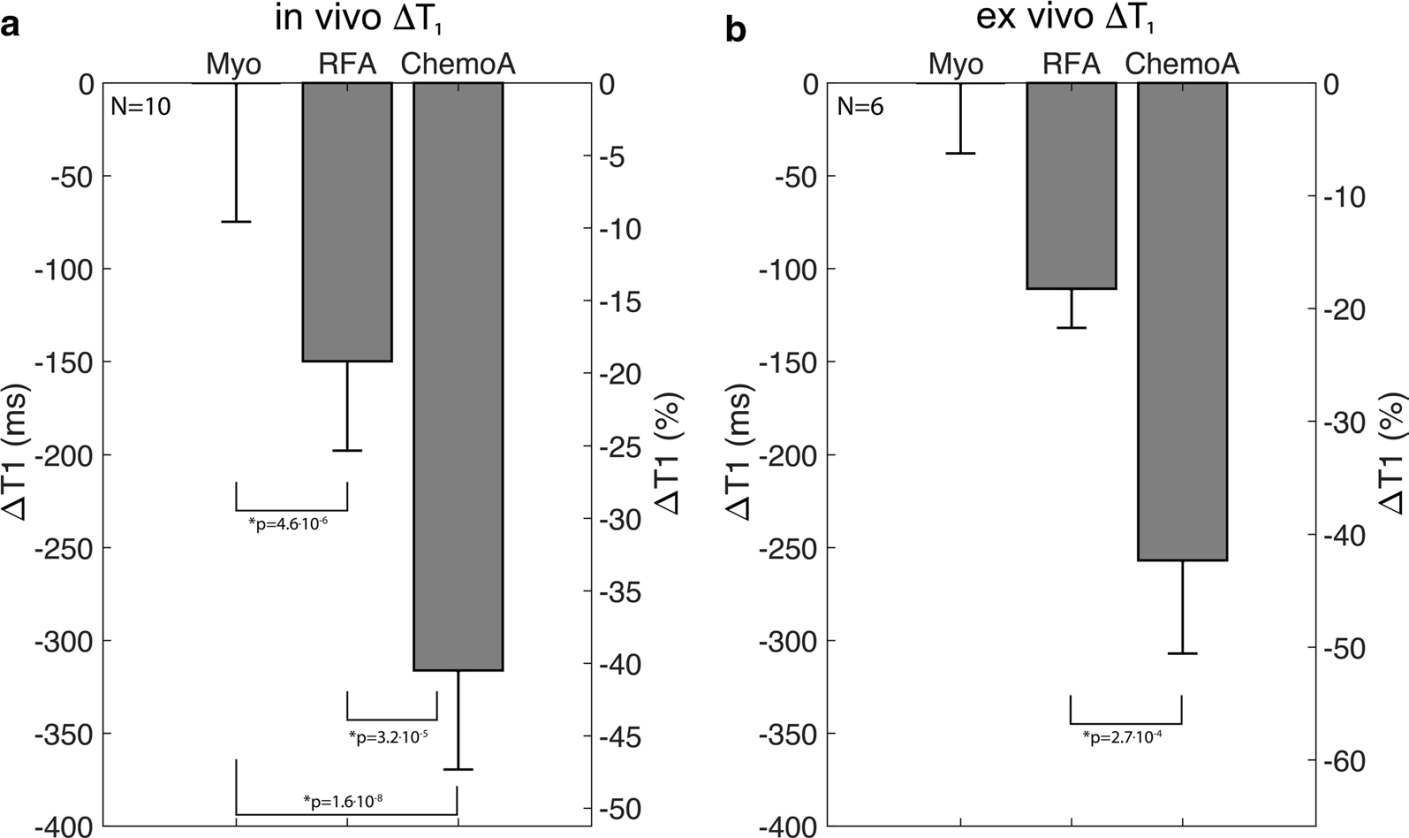
In vivo and ex vivo change in T_1_ relaxation time for RF and chemoablation lesion cores relative to myocardial T_1_ as measured on Day 0. T_1_ measured in vivo **a** demonstrates an average drop of 149 ± 49 ms (19 ± 7%) in RF ablation lesion cores and 316 ± 53 ms (40 ± 7%) for chemoablation lesions cores relative to normal myocardium. T_1_ measured ex vivo **b** demonstrates an average drop of 110 ± 21 ms (18 ± 3%) in RF ablation lesion cores and 257 ± 50 ms (42 ± 6%) for chemoablation lesions cores relative to normal myocardium. All statistical comparisons indicated significant differences (Student’s t-test, paired, Bonferroni correction). Overall, chemoablation produced 2.1 × and 2.3 × greater effect in vivo and ex vivo in myocardial T_1_ at lesion cores, respectively

Figure 3 quantifies the change in lesion T1 on the day of ablation across all ablation lesions. Chemoablation showed a larger decrease in T1 from normal myocardium compared to RF ablation. For in vivo T1 mapping, T1 decreased from 783 ± 21 ms in normal myocardium to 634 ± 66 ms in the lesion core of RF ablation and 467 ± 58 ms in chemoablation. RF ablation lesion cores had a significant drop in T1 of 149 ± 49 ms (19 ± 7%) relative to normal myocardium (p = 4.6 × 10^–6^, paired t-test, modified Bonferroni correction). Chemoablation lesions demonstrated a larger T1 drop of 316 ± 53 ms (40 ± 7%, p = 1.6 × 10^–8^). The greater drop in T1 for chemoablation compared to RF ablation was also significant (p = 4.6 × 10^–5^). Ex vivo imaging provided higher resolution and reduced partial volume averaging, but similar results for the T1 change between ablation methods. For RF ablation, the drop in T1 relative to normal myocardium was 18 ± 3%, and for chemoablation the drop in T1 was 42 ± 6%. This difference between ablation methods was also significant (p = 2.7 × 10^–4^, paired t-test).

**Fig. 4:**
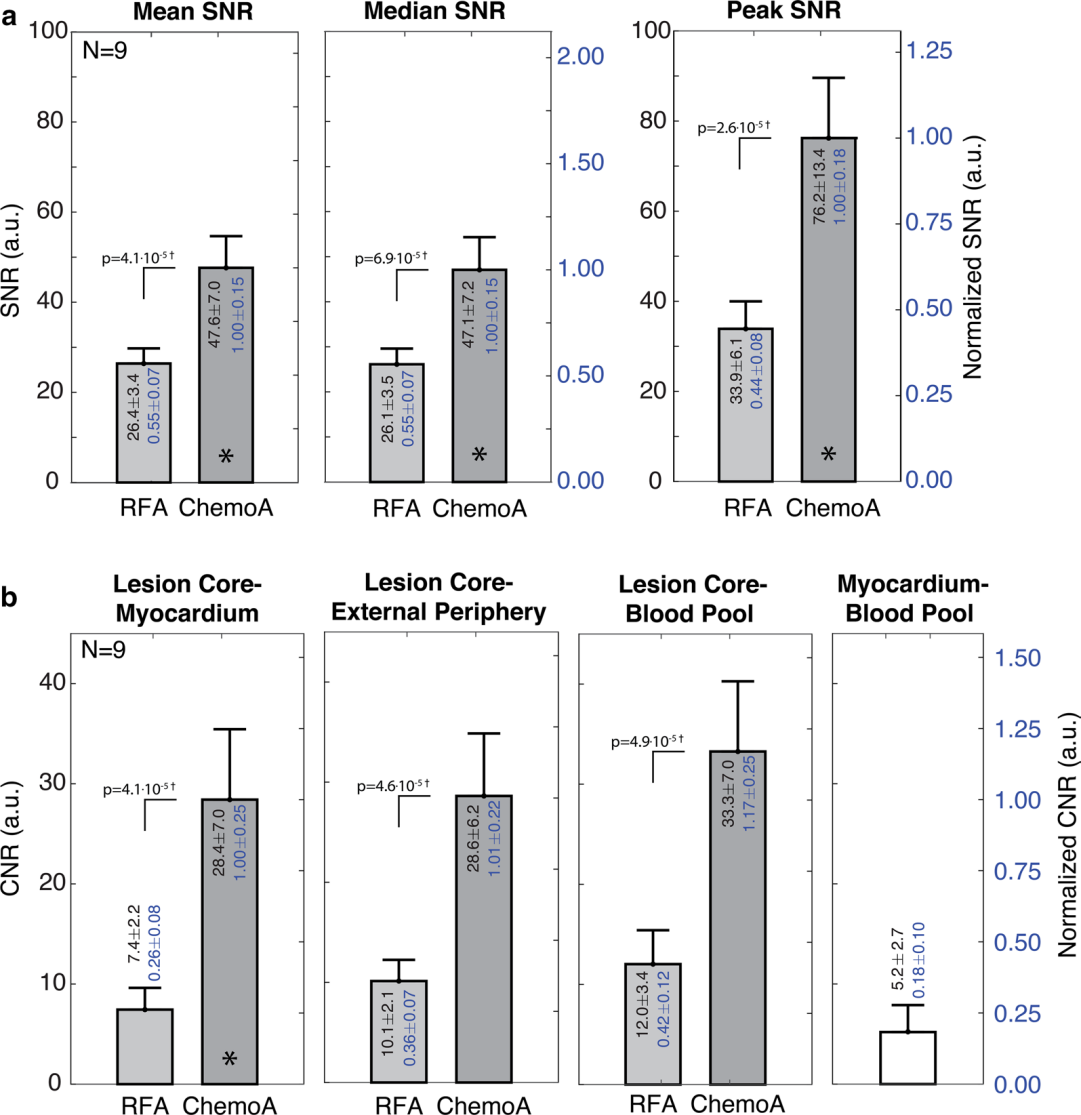
Comparison of chemoablation and RF ablation lesion signal-to-noise ratios (SNR, **a**) and contrast-to-noise ratio (CNR, **b**) obtained with 3D native contrast T1w imaging with 2RR triggering acutely on Day 0 after ablation. SNR values are normalized to the chemoablation SNR denoted by the asterisks. CNRs values are all normalized to the lesion core-myocardium CNR (*) to facilitate comparison across categories (right scale). Chemoablation lesion cores had significantly higher mean (× 1.8), median, and peak SNR than RF ablation lesion cores, resulting in improved lesion conspicuity. Chemoablation also demonstrated higher CNR with respect to myocardium (× 3.8), lesion external periphery (× 2.8), and the blood pool. RF ablation lesions had a hypointense rim, likely due to surrounding edema, that resulted in 38% higher lesion-periphery CNR than lesion-myocardium CNR. This difference was not noted for chemoablation, suggesting less surrounding edema for these lesions. Significant differences are denoted by † (Student’s t-test, paired, Bonferroni correction). As a reference, blood pool-myocardium CNR was 0.18 ± 0.10 indicating blood pool signal suppression through inversion-recovery worked well. Hence, chemoablation demonstrated significant improvement in SNR and CNRs

Figure 4a shows how the different decreases in T1 between ablation methods translated to differences in the SNR of T1w imaged ablation lesions. Both RF and chemoablation lesions had sufficient SNR for visualization. The mean, median and peak SNRs for RF ablation and chemoablation lesions, were all significantly different (paired t-tests, modified Bonferroni correction). Chemoablation had an 81% increase in mean and median SNR of the lesion core compared to RF ablation. The peak SNR for chemoablation lesions was 125% higher than RF ablation lesions (76.2 ± 13.4 vs. 33.9 ± 6.1).

**Fig. 5:**
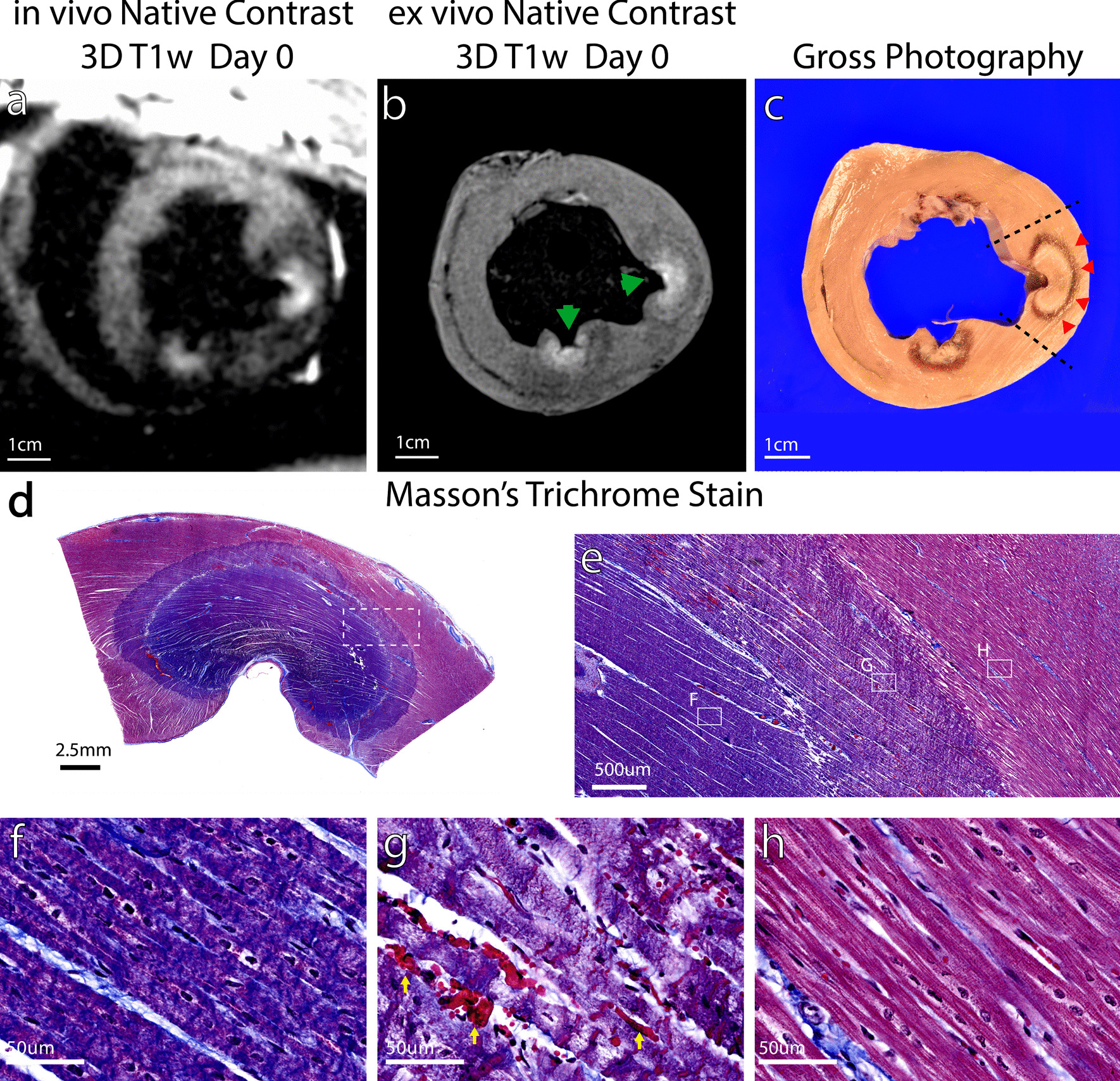
Correlation of native contrast T1w imaging to histology for RF ablation lesions (green arrows). **a** In vivo T1w imaging, **b** ex vivo T1w imaging with reduced partial volume effects, and **c** corresponding gross photography demonstrate that T1 enhancement correlates better with the pale inner core of the lesion rather than the darker outer rim of the lesion (**c**, red arrows). **d** Histology of this lesion shows the two distinct zones of necrosis indicated by shades of deeper purple trichrome staining. The dashed rectangle inset is expanded in (**e**) to focus on the lesion boundary. Solid rectangle insets are expanded to show the (**f**) inner core of coagulation necrosis, and (**g**) outer rim of mixed coagulation necrosis and thin outer band of contraction band necrosis as well as significant numbers of extravasated red blood cells (yellow arrows), before transitioning to (**h**) normal myocardium. The outer rim of RF ablation lesions (**g**) does not appear to result in T1-enhancement

Both RF and chemoablation generated significant contrast relative to the myocardium and blood pool. Figure 4b compares T1w imaged ablation lesion core CNRs relative to normal myocardium, external lesion periphery, and blood pool all of which produced statistically significant differences (paired t-tests, modified Bonferroni correction). After scaling for differences in acquisition parameters, across all three CNRs displayed, chemoablation lesions generated higher contrast than RF ablation lesions (2.7×–3.8×). Ablation lesion to myocardium CNR was ~ 3.8 × higher for chemoablation compared to RF ablation. For RF ablations, the lesion core to external periphery CNR was 36% greater than lesion core to myocardium CNR. Figures 1b, 2b and 5a, show that RF ablations were surrounded by a hypointense rim. This likely reflects the distribution of edema surrounding the lesion, with edema’s longer T1 resulting in hypointensity relative to normal myocardium. For chemoablation, the lesion core to myocardium CNR was very similar to the lesion core to lesion periphery CNR suggesting chemoablation has less edematous border surrounding lesions. The myocardium-blood pool CNR is reported for comparison.

Figure 5 shows RF ablation correlations between in vivo T1w imaging (Fig. 5a) and ex vivo T1w imaging (Fig. 5b), gross pathology (Fig. 5c), and histology Masson’s trichome histological stain on ablation Day 0. In vivo T1w imaging shows a bright lesion core surrounded by a hypointense rim, that is also noted on ex vivo T1w imaging. Gross pathology shows the RF ablation lesions have a pale inner lesion core and a darker outer lesion rim. Both the core and rim are composed of necrotic tissue as shown in histology (Fig. 5d–h). The inner lesion core is composed of uniform coagulation necrosis (Fig. 5f). The outer rim (Fig. 5g) is composed of mixed coagulation necrosis and a much larger number of extravasated red blood cells (yellow arrows) that likely give the tissue its darker coloration in gross pathology. At the boundary between the lesion and normal myocardium (Fig. 5e inset g vs. h), a thin region of contraction band necrosis can also be discerned. From in vivo CMR, it is difficult to determine whether or not lesion enhancement corresponds to the entire necrotic lesion. Ex vivo CMR, with much higher resolution and SNR, more clearly visualizes components of the lesion boundary. The enhancing core on imaging appears to correspond to the inner necrotic lesion core on pathology and histology. A rim of diminished enhancement on ex vivo imaging corresponds to the outer necrotic lesion rim. This supports prior findings that for RF ablation, T1w imaging underestimates necrotic lesion dimensions and volume [17]. Additional imaging to pathology correlation is provided in Additional file 2: Figure S1.

**Fig. 6:**
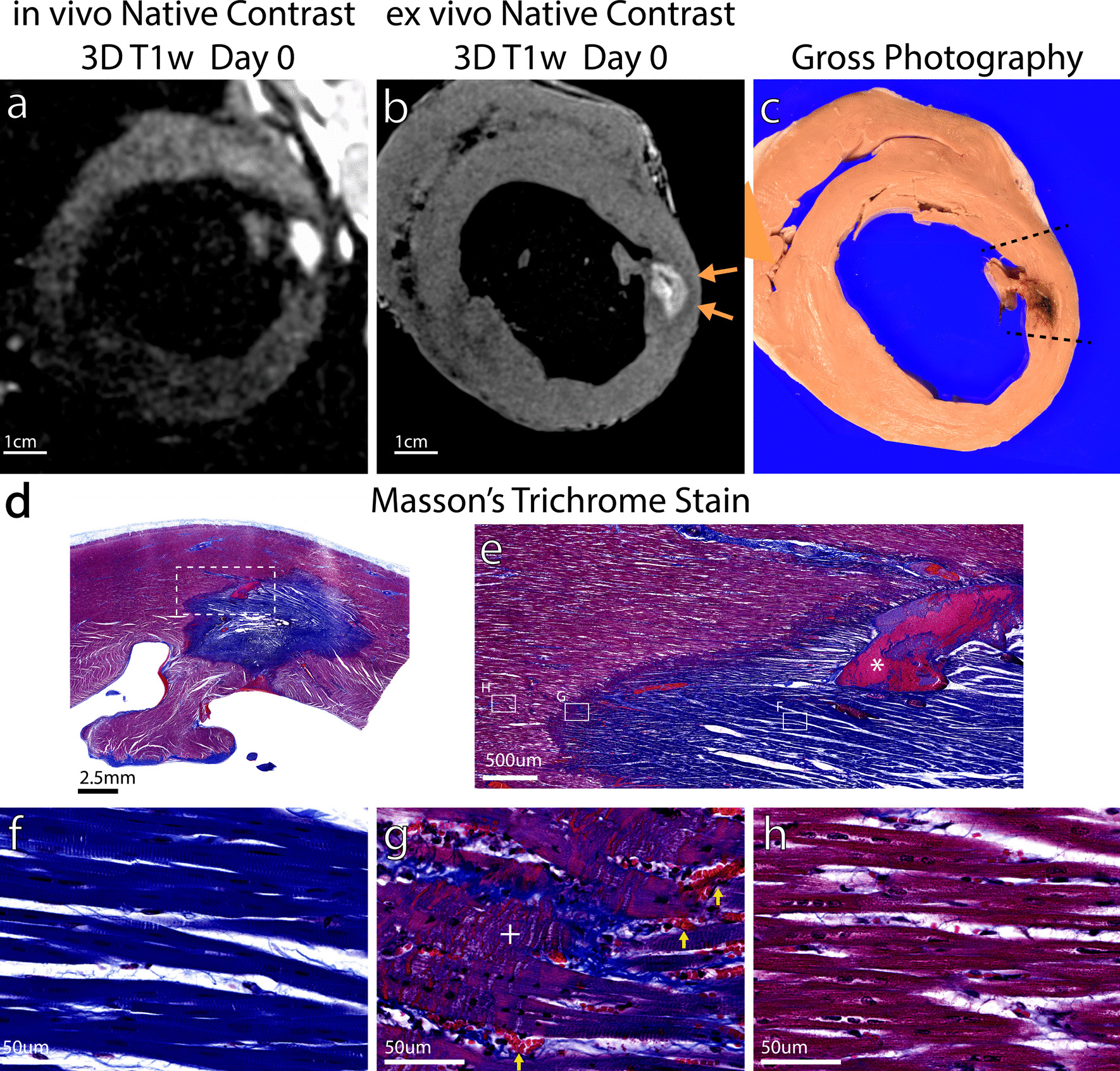
Correlation of native contrast T1w imaging to histology for chemoablation (orange arrows). T1-enhancement and necrosis correlate well, making assessment of chemoablation lesion extent reliable with T1w imaging. Contrast in in vivo (**a**) and ex vivo (**b**) T1w imaging depends on reduction of T1 relaxation time. The region of T1w enhancement correlates well with the pathology lesion (**c**) and necrotic lesion with deeper purple trichrome stain on histology (**d**). Closer observation of the lesion periphery (**e**) reveals a core of coagulation necrosis (**f**) with a small transition band composed of contraction band necrosis (**g**, + mark) and significant number of extravasated red blood cells (**g**, yellow arrows) before reaching normal myocardium (**h**). Compared to RF ablation, for chemoablation the entire extent of necrosis observed under histology correlates better with the enhancing region on T1w imaging. The asterisk (*) in (**e**) marks the likely needle track with hemorrhage and coagulated red blood cells

Figure 6 shows chemoablation correlations between in vivo T1w imaging (Fig. 6a), ex vivo T1w imaging (Fig. 6b), gross pathology (Fig. 6c) and Masson’s trichrome histological stain on ablation Day 0. In vivo T1w imaging and ex vivo T1w imaging display very similar dimensions for the lesion. In vivo imaging also shows a smaller hypointense rim suggesting reduced edema compared to RF ablation. Gross pathology shows a heterogeneous lesion core with outer dimensions that correspond well to in vivo and ex vivo imaging. Staining (Fig. 6d–h) demonstrates a lesion core composed of coagulation necrosis (Fig. 6f), a narrow peripheral rim with significant presence of ectravasated red blood cells (g, yellow arrows), and a very narrow region of contraction band necrosis at the boundary of the lesion with normal myocardium (Fig. 6h). This pattern is similar to that observed with RF ablation lesions (Fig. 5), though the peripheral rim region is much narrower. The void left by the needle injection is filled with coagulated red blood cells (asterisk in Fig. 6e). Additional imaging to pathology correlation is provided in Additional file 2: Figure S1.

**Fig. 7:**
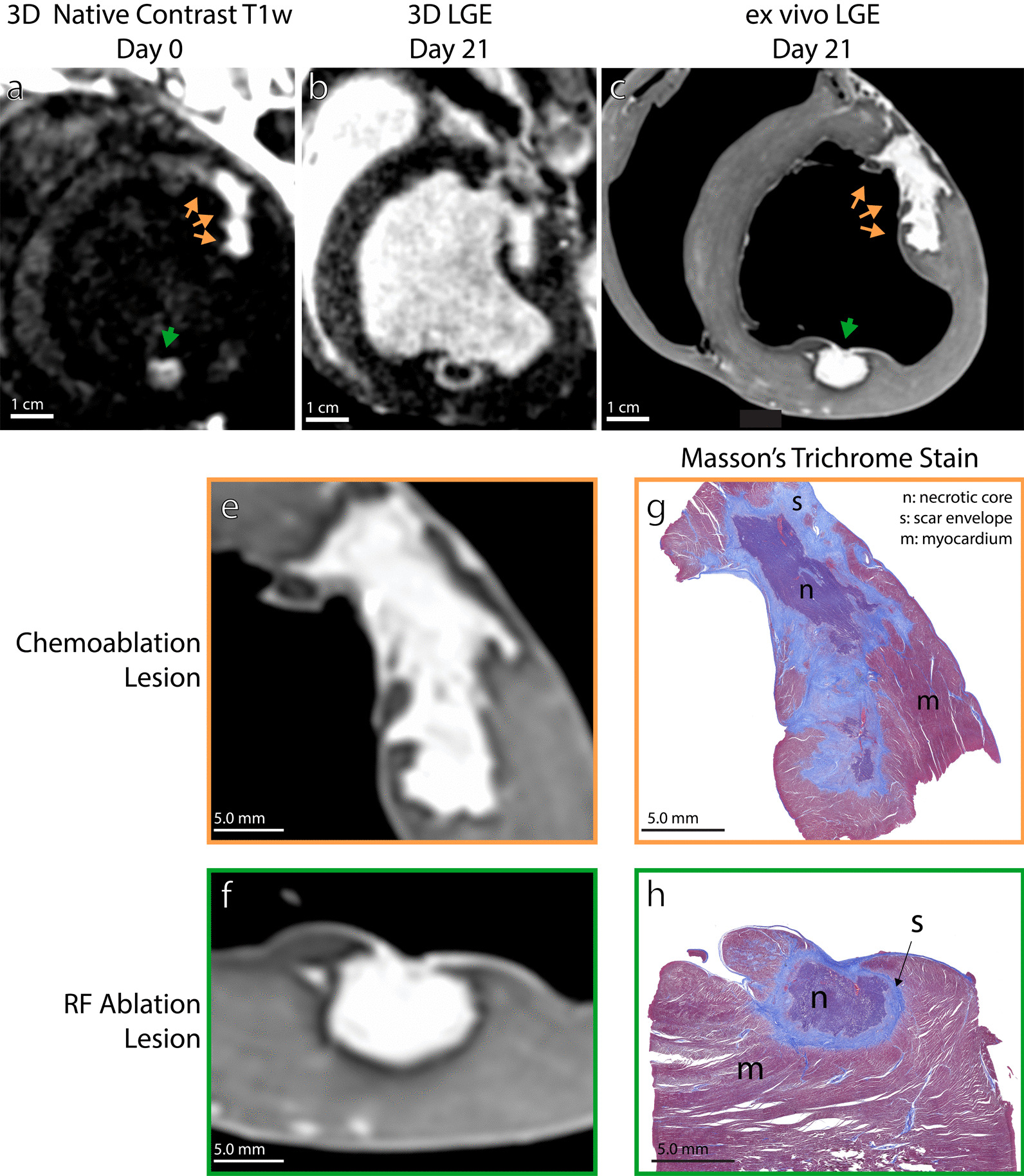
Acute and chronic post-ablation imaging for evaluating long term lesion size. The enhancing boundaries for RF ablation lesions (green), and chemoablation (orange) are well visualized by (**a**) acute native contrast T1w imaging (Day 0), and (**b**) chronic late gadolinium enhancement (LGE) on Day 21. Both show excellent correspondence to ex vivo LGE (**c**,**e,f**) and trichrome stain histology (**g**,**h**) performed on Day 21. These results support the hypothesis that T1w imaging on the day of ablation describes long-term lesion size. Histology shows that encapsulation and the wavefront of healing progress inwards as collagen and scar tissue (s, light blue) replace resorbed necrosis

Figure 7 compares acute post-ablation T1w imaging (Day 0), chronic LGE imaging on Day 21 after ablation, and Day 21 histology. In vivo T1w imaging shows RF ablation (green arrows) and chemoablation (orange arrows) can be visualized acutely (Fig. 7a). After 3 weeks, in vivo LGE imaging shows both RF ablation and chemoablation lesions are composed of an outer hyperenhancing region and central hypointense core (Fig. 7b). Lesion boundaries are more distinct on ex vivo LGE imaging (Fig. 7c). The region of in vivo LGE enhancement corresponds to the outer region of fibrotic scar on histology (labeled *s* on Fig. 7g, h). The hypoenhancing lesion core in in vivo LGE corresponds on histology to the area of residual necrosis that has not yet undergone fibrotic replacement (labeled *n* on Fig. 7g, h). Lesion boundaries are more distinct on ex vivo LGE imaging (Fig. 7c). The lesion boundaries on Day 21 ex vivo LGE match well with necrotic lesion extent on histology for both chemoablation (Fig. 7e,g) and RF ablation (Fig. 7f,h). These data illustrate the utility of acute T1w imaging and chronic LGE imaging to indicate the extent of permanent fibrotic ablation lesions.

**Fig. 8:**
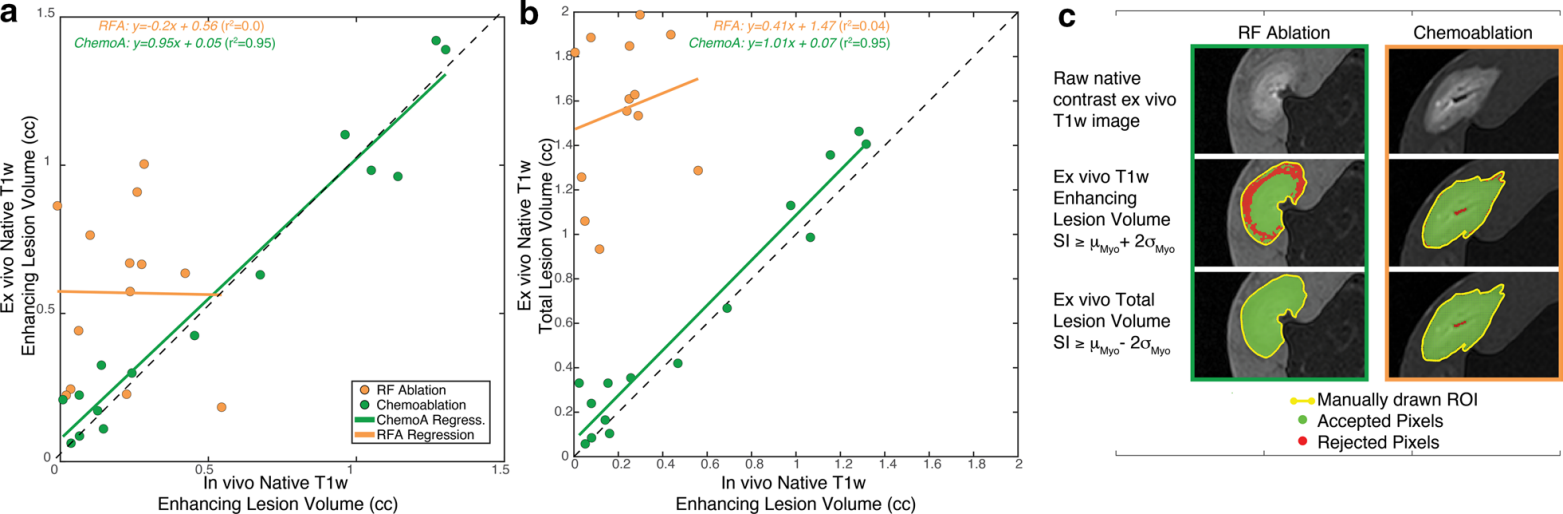
Regressions between lesion volumes measured on Day 0 in vivo and reference volumes from ex vivo measurements under ideal conditions determine whether the T1w enhancement visualized in vivo is predictive of lesion size. **a** Correlation of enhancing lesion volumes from in vivo T1w imaging with matching ex vivo enhancing volume indicates how well in vivo CMR can visualize lesion enhancement. **b** Correlation of enhancing lesion volume from in vivo T1w imaging with matching ex vivo total lesion volume demonstrates whether in vivo T1w imaging is indicative of lesion extent. **c** Pictorial description of thresholds used to calculate the lesion volumes from ex vivo native contrast T1w CMR. Overall, chemoablation lesions demonstrate significantly better correlations

To further examine the ability of T1w imaging at low field to describe the extent of necrosis, three correlations are considered. In Fig. 8a, in vivo and ex vivo T1w enhancing lesion volumes on Day 0 for 13 (33%) RF ablation and 15 (75%) chemoablation non-overlapping lesions. Chemoablation lesions show excellent correlation while RF ablations show no correlation. Comparison between T1w enhancement volume and total lesion volume (Fig. 8b) demonstrates how well in vivo imaging can predict final extent of necrosis. Again, T1w enhancement from chemoablation is an excellent predictor of total lesions volume while for RF ablation, the volumes have little correlation. Figure 8c includes a pictorial description of the thresholds used to determine both the T1w enhancing volume and the total lesions volume. In Additional file 3: Figure S2, the T1w lesion volume and total lesions volume, both measured from ex vivo imaging, are compared. A larger fraction of chemoablations enhances (89 ± 11%, range 60–98%) than for RF ablation (36 ± 19%, range 13–81%).

**Fig. 9:**
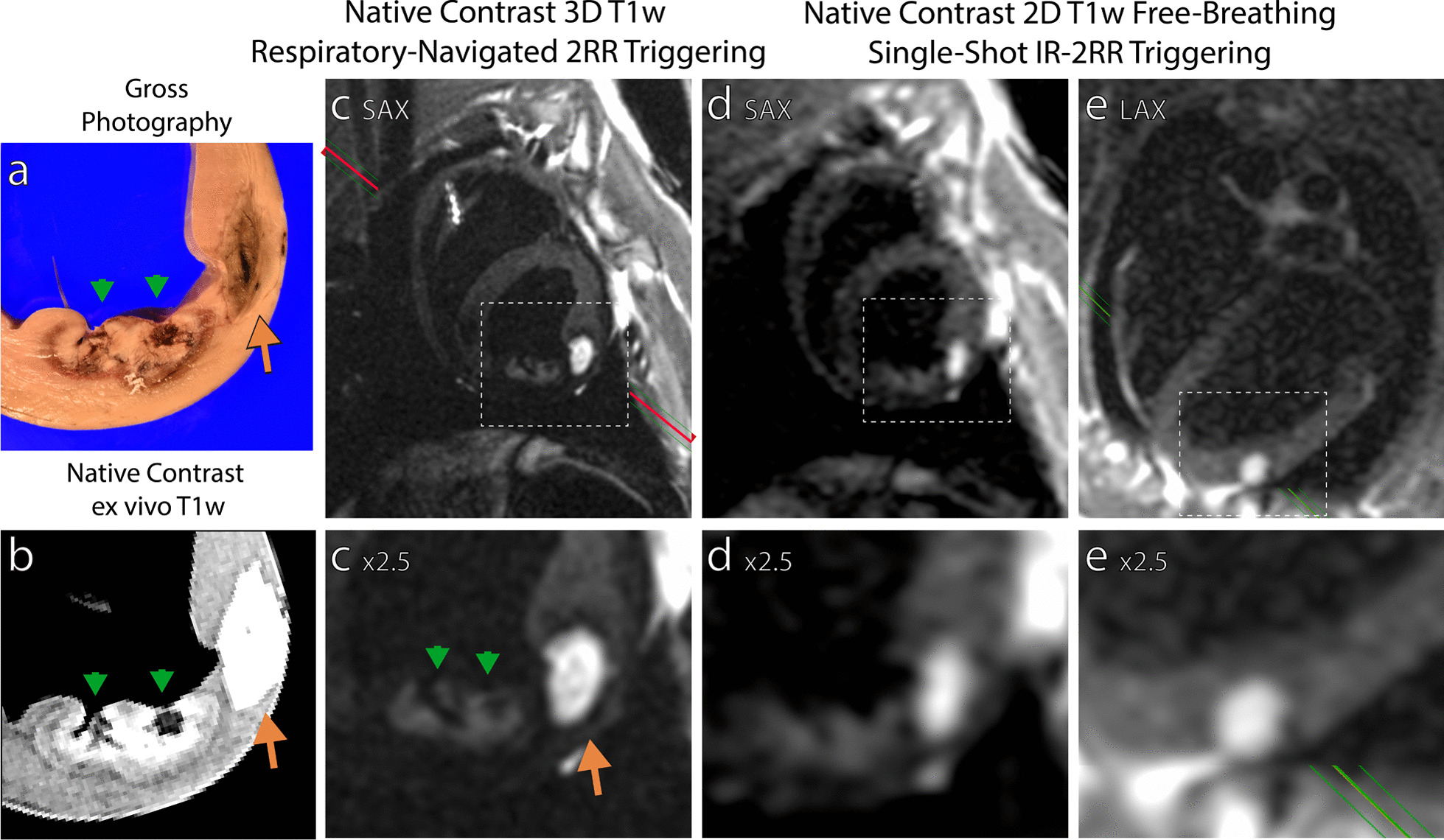
Visualization of ablation lesions with single-shot native contrast imaging, suitable for monitoring lesion placement during an ablation procedure. **a** Gross pathology and **b** ex vivo imaging of two RF ablation lesions (green arrows) and one chemoablation lesion (orange arrow), **c** in vivo 3D navigator gated T1w imaging shows good correspondence to the lesion core regions on ex vivo assessment. The chemoablation lesion has higher contrast compared to RA ablation lesions. **d**,**e** in vivo single-shot imaging with 2-RR triggering in both short axis (SAx) and horizontal long axis (LAx) corresponds well to in vivo 3D imaging This suggests visualization of lesions immediately after and likely during formation is feasible with 2-RR triggering, particularly for higher contrast chemoablation lesions. Additional files 4 and 5: Video S1 display a time series of the images (**d**)

While 3D imaging permits assessment of overall ablation lesion distribution at the end of an ablation procedure, imaging time limits its use for monitoring adequate placement of individual ablation lesions. Figure 9 illustrates the ability to visualize ablation lesions using near-real-time 2D single-shot IR imaging with 2-RR triggering in a 0.55 T CMR scanner. Though RF ablations can be visualized in the single shot images (Fig. 9d, green arrows), chemoablations are more conspicuous due to the larger change in T1 (Fig. 9d, orange arrows). A temporal series of the single-shot T1w lesion images is provided Additional files 4 and 5: Video S1.

## Discussion

This work shows that native contrast T1w CMR at 0.55 T can assess acute ablation lesions in the heart. Native contrast imaging can visualization morphological characteristics of myocardial RF ablation lesions but is limited by SNR. Acetic acid chemoablation by direct intramyocardial injection is an attractive experimental therapy producing lesions that can be accurately assessed at low field. Native contrast imaging of ablation lesions offers the ability to assess delivered therapy without the use of Gd-based contrast agents which limit intraprocedural feedback as they can only be used once or twice during intervention and tend to overestimate the extent of necrosis [11, 17].

Ablation lesion imaging at low field could simplify clinical translation of CMR guided cardiac electrophysiology procedures by reducing the concern for RF heating of interventional devices. Because RF heating is proportional to the square of field strength, the ability to perform lesion assessment 0.55 T compared to standard 1.5 or 3 T enables use of some commercially available guidewires and simplifies heating-safe design requirements for other electrophysiology catheters and sheaths [25, 33]. However, lower field results in lower polarization and reduced image SNR. We found that SNR from electrocardiogram (ECG) and respiratory navigator gated 3D imaging and ECG gated 2D imaging over 2 heartbeats was sufficient to permit lesion visualization. This was particularly true for acetic acid chemo-ablation which exhibited around ~ 3.7 fold greater native T1 contrast than RF ablation.

Accurate characterization of injection chemoablation lesions could address the current limitations of RF in ablating arrhythmogenic tissue deeper within the myocardium which contributes to poorer outcomes in ablation of VT in non-ischemic forms of cardiomyopathy [2]. In particular, inadequate ablation of arrhythmogenic substrate is a major contributing factor to high recurrence of VT after ablation [2, 34]. The ability to predict the location and extent of permanent ablation is currently limited. Native contrast T1w CMR has shown recent promise by accurately predicting the extent of necrotic RF ablation lesions within minutes of ablation [20, 35]. Needle injection of acetic acid can be targeted to ablate deeper myocardial structures, and we have found the resulting lesions can also be detected by native contrast T1w CMR.

Since the goal of lesion assessment is to predict the extent of permanent tissue injury, this work sought to carefully characterize the correlation between lesion enhancement on imaging and tissue necrosis. To accomplish this, we first established the correlation of native T1 contrast between in vivo and higher-resolution ex vivo imaging. We found the percent relative T1 shortening for both acetic acid chemoablation and RF ablation relative to normal myocardium was similar between in vivo and ex vivo native contrast T1 mapping. This similarity provided the rationale for comparing contrast between in vivo and ex vivo T1w imaging. Structural comparisons were then made between in vivo and high-resolution ex vivo lesion imaging and histology.

### Native contrast CMR visualization of RF ablation necrosis

The mechanism of tissue necrosis caused by RF ablation is thermal coagulation of myocytes as temperature is raised above around 50 °C [22]. The dominant mechanism of native T1 contrast generated by RF ablation is thought to be oxidation of ferrous to ferric iron in myoglobin and hemoglobin within the zone of thermal necrosis [20, 24, 35, 36]. As shown in this work, the zone of native T1 contrast on in vivo and ex vivo imaging does not encompass the entire necrotic lesion observed on histology. RF ablation lesion necrosis on histology was composed of two zones: an inner zone of coagulation necrosis, and an outer zone of mixed coagulation necrosis in addition to significant presence of intact red blood cells (Fig. 5). Likely, the temperature rise in the periphery of the lesions is sufficient to cause necrosis but insufficient to trigger the mechanisms by which T1 contrast is generated (i.e. formation of metglobins [20, 35] or generation of protein crosslinking). This disparity of the underlying mechanisms merits further study.

The T1w enhancement reflects the inner core of necrosis on both in vivo and ex vivo imaging, resulting in underestimation of total lesion volume. These separation between the peripheral and core areas may not have been previously observed as clearly in ex vivo imaging (Figs. 5b, 8b and Additional file 2: Figure S1, Additional file 3: Figure S2) due to Gd-based agents (e.g. [17, 35]…), since the non-enhancing peripheral zone would be automatically included in Gd-based measurement of lesion volume or dimensions. The difference in these two acquisitions is clear when comparing Figs. 5b (Gd−) and 7F (Gd +). In vivo, the T1w contrast of RF ablation lesions was accentuated by a hypointense surrounding region between the enhancing lesion and normal myocardium. This region corresponds with the extent of enhancement on T2w imaging and is likely attributable to tissue edema with increased T1. (Fig. 1 and Additional file 2: Figure S1).

Prior in vivo studies have also shown underestimation of lesion dimensions by T1w imaging but demonstrated good correlation between imaging and pathology. However, Fig. 8a shows little correlation between RF ablation lesions in vivo and ex vivo. The most likely contributing factor is the lower SNR and CNR at low field. Also, our acquisition used lower through-plane resolution (3.0 mm) than prior studies, (e.g. [21]) which results in more partial volume averaging. In addition, RF ablation lesions in this study were generated with higher power with the intent to increase lesion dimensions. However, the increased tendency for tissue overheating contributed to heterogeneous lesions with more complex geometries, including some with cavitation at the core.

### Native contrast CMR imaging of acetic acid chemoablation necrosis

Acetic acid chemoablation also causes tissue coagulative necrosis, and if high enough in concentration, liquefaction necrosis [37]. We have observed that not all chemoablation causes T1 shortening, for example ethanol chemoablation. The mechanism of native T1 contrast with acetic acid ablation is undergoing further study. Compared to RF ablation, the zone of chemoablation T1 shortening on in vivo and ex vivo imaging appears more closely correlated with the region of tissue necrosis on histology. This is likely related to the lesion being more completely composed of coagulation necrosis with a much thinner peripheral rim of mixed necrosis and intact red blood cells compared to RF ablation lesions (Figs. 5,6). The twofold greater T1 shortening caused by chemoablation results in greater lesion enhancement compared to RF ablation. As with RF ablation, a region of hypoenhancement is observed around the periphery of chemoablation lesions and likely reflects tissue edema. However, the extent of hypoenhancement is less than for RF ablation. This suggests recurrence of arrhythmia due to recovery of transient injury and edema may be less of an issue for chemoablation but this hypothesis requires further study. The combination of high lesion contrast, good correlation of lesion contrast with the extent of necrosis, and the potentially limited extent of transient tissue injury and edema, appears to make acetic acid chemoablation a favorable method for performing CMR-guided ablation.

### Correlation of lesion volumes: in vivo vs. ex vivo imaging

Three-dimensional high-resolution native contrast ex vivo imaging is very accurate for the measurement of necrotic lesion volumes as shown by correlation with histology and can therefore act as a reference for in vivo measurements. As shown in this work, chemoablation lesions display higher SNRs and CNRs than RF ablation lesions due to a larger decrease in T1. Hence, in vivo these lesions are less affected by partial volume averaging as peripheral pixels display sufficient enhancement on T1w imaging resulting in excellent correlation between ex vivo lesion volumes and in vivo T1w enhancing volume (Fig. 8a,b).

RF ablations on the other hand, display little correlation between in vivo and ex vivo results. This outcome could result from in vivo lesions with lower CNRs being more susceptible to partial volume effects compounded by smaller enhancing volumes and more heterogeneous lesions due to the presence of coagulum, and encroachment of peripheral isointense and hypointense zones of the lesion that are visible on ex vivo imaging.

As shown in Additional file 6: Figure S3, on average, 89% of chemoablation lesion volumes were T1w enhancing on ex vivo imaging when using a (μ_Myo_ + 2σ_Myo_) threshold (r^2^ = 0.96). RF ablations on the other hand, display little correlation between in vivo and ex vivo results. On average, 36% of an RF ablation lesion total volume is enhancing on ex vivo imaging (Additional file 6: Figure S3).

These in vivo to ex vivo lesion comparisons are based on defining lesion enhancement as μ_Myo_ + 2σ_Myo_. By this definition, on average 89% of the ex vivo chemoablation lesion volume enhances compared to 36% of the ex vivo RF ablation lesion volume (Additional file 6: Figure S3). The Fig. 8d displays the effects of the threshold selection for both chemoablation and RF ablation lesions. The high SNR of chemoablation lesions results in few peripheral pixels not being considered enhancing by our initial threshold. Only hypointense pixels that correspond to blood at injection sites or liquefaction necrosis from 100% acetic acid are excluded. However, for RF ablations, the non-enhancing peripheral region reduces the volume identified as necrotic both in vivo and ex vivo. Potentially, using a more lenient threshold for T1w enhancement (e.g. 1.0 or 0.5 myocardial standard deviations above the myocardial mean) could result in better correlation at the risk of losing specificity for necrosis and further work should clarify this. Previous work at higher field strengths has demonstrated better correlation between in vivo and ex vivo results, which suggests that the reduced SNR could present a challenge for RF ablation at lower field strength [17]. Note that our analysis was based solely on non-overlapping lesions observed on Day 0 imaging to avoid confounding between core T1w enhancement and peripheral non-enhancement.

### Application of native contrast T1w CMR for monitoring ablation procedures

3D native contrast T1w CMR has favorable properties for ensuring completed ablation at the end of ablation procedures. Pathways of viable myocardium passing through myocardial scar are the typical substrate for VT in patients with structural heart disease. A clinically relevant component of this arrhythmogenic substrate appears detectable by 3D CMR with ~ 1.5 mm^3^ resolution [38]. Recent reports suggest that targeting CMR identified arrhythmogenic substrate results in favorable VT ablation outcomes compared to conventional ablation guided by electrophysiologic measurements [39]. From prior work evaluating the T1w CMR appearance of RF ablation within scar, ablated myocardium but not pre-existing scar enhances following ablation [17, 20]. 3D T1w CMR performed at the end of an ablation procedure could be used to confirm corridors of viable myocardial within scar have been adequately ablated before ending the procedure.

Based on the currently high recurrence of VT after ablation, multiple ablation attempts may be needed to adequately ablate the arrhythmogenic substrate. In this setting, ablation performed within the CMR scanner using real-time native T1w CMR guidance is attractive because lesion assessment can be more easily repeated after additional ablation is performed until complete ablation is confirmed. In addition to reducing the need to move the patient for lesion reassessment, catheter position from real-time CMR is inherently registered to lesion imaging, which avoids the need for time consuming and error prone registration of lesion imaging to conventional catheter position tracking systems.

However, the ability to repeat 3D imaging during a procedure is restricted by relatively prolonged imaging times. Rapid 2D imaging using 2-RR triggering (Fig. 9) to assess lesion placement and extent could be an attractive method to monitor the proper positioning of individual lesions before 3D confirmation of adequate ablation at the end of a procedure. Though 2D imaging sacrifices resolution, particularly when imaging at low field, this study found that both RF and chemoablation lesions could be detected by 2D native contrast single-shot T1w CMR imaging with two heartbeat triggering. Compared to RF ablation, acetic acid chemoablation lesions are more readily visualized during rapid 2D imaging on a 0.55 T scanner as expected from their higher T1w contrast.

### Limitations

The 3D images presented in this work required long scan times. Acquisitions did not take advantage of undersampling with parallel imaging or advanced reconstruction, two prevalent tools for scan time reduction. There were two reasons for this: (1) the coils available in the prototype low field system are yet to be optimized (e.g. element size, number of elements, etc.) resulting in steep penalties for parallel imaging, and (2) to facilitate SNR analysis. Further improvement in phased arrays is expected to enable contemporary undersampling methods such as parallel imaging and compressed sensing to reduce scan time. Scan time could also be reduced by using a single heartbeat for recovery to half scan duration. However, this is likely to make image contrast more dependent on heart rate and rhythm regularity [20]. Saturation recovery could address this issue at the expense of lesion-myocardium contrast and blood-pool-myocardium contrast. Evaluation of these methods for reducing scan time merits further study.

We hypothesized higher concentration of acetic acid would produce lesions with larger volume, however further work is required to accurately relate variables like concentration and injection volume to lesion volume. A number of variables contribute to the formation of chemoablation lesions and can affect the accuracy of injection targeting. Injection material preferentially follows tissue planes along paths of lower resistance to flow. This resistance varies over time with the beating heart. Lesion growth appears limited once injection material finds a path to the endocardial or epicardial surface which provides a lower resistance path than intramyocardial infiltration. From our experience in this study, it appears starting injection a few millimeters away from the endocardial surface and epicardial surface and injecting material slowly (over 1–2 min) increases lesion size. However, procedure guidance using X-ray fluoroscopy and ICE lacked the ability to accurately visualize needle depth. We anticipate the ability to monitor lesion formation using real-time CMR will be useful for accurately targeting chemo ablation lesions.

The DESPOT sequence used to measure ex vivo T1 in the cohort of animals killed of Day 0 (without added Gd-based contrast agents) resulted in residual coherences that biased T1. Retrospective correction based on phantom calibration, allowed for a better estimate of ΔT1. Nevertheless, the relative ΔT1% still provided an idea how the two ablation techniques differed, and the matching results between ex vivo and in vivo ΔT1 indicates corrections are appropriate.

With only two time points, it was not possible to discern if the two lesion types healed equally or whether coagulation necrosis generated through RF or chemoablation resorbed at the same rate during the healing wavefront. Though Fig. 2 indicates a larger decrease in chemoablation lesion volume relative to RF ablation lesion volume, this could be due to differences in imaging plane and cannot be ascribed to differences in healing patterns without further work. Preliminary analysis of scar observed in histological data (Masson’s trichrome stain) indicates that both lesion types are surrounded by layers of scar tissue of similar thickness.

## Conclusions

Accurate identification of tissue necrosis is the goal of ablation lesion characterization in CMR. This work demonstrates that native contrast T1w imaging allows for visualization of cardiac necrosis produced by both RF ablation and acetic acid chemoablation on a low-field CMR platform. The change in T1 produced by chemoablation was higher than that with RF ablation, improving visualization of necrosis and resulting in better estimation of lesion volumes. By comparison, RF ablation lesions exhibit a larger peripheral region of overlapping necrosis and hemorrhage with decreasing changes to T1 which leads to underestimating the extent of necrosis. Transcatheter chemoablation represents an attractive method for targeted cardiac tissue destruction, particularly when using low-field CMR.

## Supplementary Information

**Additional file 1:** Additional methods

**Additional file 2: Figure S1.** The hypointense rim on native contrast T1w CMR corresponds well with T2w imaging depicting peripheral edema. In vivo (**a**) T2w imaging with manually segmented region of edema. In vivo (**b**) native contrast T1w imaging shows a clear hypointense boundary surrounding the RF ablation (green arrow). The region of edema from T2w imaging is superimposed and aligns well with hypointense region. Chemoablation lesions (orange arrow) do not exhibit the same peripheral hypointensity. (**c**) Ex vivo native contrast T1w CMR clearly depicts the extent of both RF ablation and chemoablation lesions.

**Additional file 3: Figure S2.**Visual correlation of RF ablation and chemoablation lesions acutely on Day 0 after ablation using gross photography, native contrast CMR and histological stains. Gross photography (**a**) compares very well with CMR (**b**) as shown by the overlay of the two (**a** +** b**). RF ablation (green arrows) and chemoablation (orange arrows) lesions are clearly seen including the boundary between T1w enhancing and non-enhancing portions of RF ablation lesions. This boundary can also be visualized on histological stains (**c**, **d**). Chemoablation lesions do not exhibit a significant non-T1w enhancing peripheral zone. The areas of necrosis can be visualized as purple in Masson’s Trichrome stain, while small pockets of blood can be most easily visualized in the H&E stain. The hypointense cavity within the septal RF ablation lesion is due to a “steam-pop” resulting from tissue overheating. Overall, there is excellent correlation between areas of T1w enhancement and the inner portion of the necrotic core of lesions.

**Additional file 4: Video S1**. Left: static frame of 3D respiratory navigator-gated T1w imaging with long TI and 2-RR triggering. Right: single-shot images acquired during free breathing also with long TI and 2-RR triggering. The images on the right are acquired every other heartbeat to maintain blood pool-myocardium contrast. Two RF ablations and one chemoablation are visualized though the chemoablation lesions is significantly more conspicuous. Images match those displayed in Fig. 9.

**Additional file 5: Video S1.**

**Additional file 6: Figure S3.**Comparing T1w enhancing volume and total lesions volume both from native contrast ex vivo imaging measures the fraction of each lesion that enhances. On average across all non-confluent lesions 89 ± 11% and 36 ± 19% of total lesion volume enhances for chemoablation and RF ablation, respectively.

## Data Availability

The datasets generated during and/or analyzed during the current study are available from the corresponding author on reasonable request.

## Abbreviations

2D: Two dimensional
3D: Three dimensional
bSSFP: Balanced steady-state free precession
CMR: Cardiovascular magnetic resonance
CNR: Contrast-to-noise ratio
CT: Computed tomography
DESPOT: Driven equilibrium single pulse observation of T1
EGE: Early gadolinium enhancement
GBCA: Gadolinium-based contrast agent
Gd: Gadolinium
GRAPPA: Generalized autocalibrating partially parallel imaging
H&E: Hematoxylin and eosin stain
ICE: Intra-cardiac echocardiography
IR: Inversion recovery
LAX: Long axis
LGE: Late gadolinium enhancement
LV: Left ventricle/left ventricular
PET: Positron emission tomography
RF: Radiofrequency
ROI: Region of interest
RV: Right ventricle/right ventricular
SAR: Specific absorption rate
SASHA: Saturation recovery single-shot acquisition
SAX: Short axis
SPGR: RF-spoiled gradient recalled echo
SNR: Signal-to-noise ratio
T1w: T1-weighted
T2w: T2-weighted
TE: Echo time
TI: Inversion time
TR: Repetition time
T_SAT_: Saturation delay time
TWILITE: T1-weighted inversion recovery imaging with a long inversion time
VT: Ventricular tachycardia
VF: Ventricular fibrillation
