# Supplementary material for: Native contrast visualization and tissue characterization of myocardial radiofrequency ablation and acetic acid chemoablation lesions at 0.55 T
Source: J Cardiovasc Magn Reson. 2021 May 6;23:50. doi: 10.1186/s12968-020-00693-1 (PMC8101152; doi:10.1186/s12968-020-00693-1)
Supplement: Supplementary file 1 — Additional file 1: Additional methods [file 12968_2020_693_MOESM1_ESM.pdf]

Additional Methods

DESPOT T1 Mapping Correction

The DESPOT mapping sequence underestimated the T1 values measured from *ex vivo* tissues, an issue likely resulting from unspoiled coherences from insufficient spoiling of the SPGR sequence and relatively short TR (30 ms). To determine if an appropriate correction existed between measured T1 values and actual values, the T1MES phantom (30) was scanned with the same 3D DESPOT sequence used to quantify T1 in *ex vivo* with TR$\in$ [30, 40, 50] ms. The latter two TRs were acquired to confirm findings and demonstrate that the effects decreased as TR increased.

As a reference, a series of IR images were acquired with a single-echo IR gradient echo sequence with adiabatic excitation. TI ranged from 10 ms to 8 sec in 16 steps. ROIs were drawn on the different vials of the phantom on both datasets in Osirix (Pixmeo SARL, Bernex, Switzerland) and exported into MATLAB (Mathworks, Natick, MA, USA) were data were fit to extract T1. Regression analysis between the measured DESPOT and IR reference T1 values for vials with $T1_{DESPOT}$<1000 ms revealed that both 1st and 2nd order polynomials provided reasonable correction. The relationship between the two datasets are show in in the following equations:

$$T1_{corrected} = 0.751 \cdot T1_{DESPOT} - 32.5 \; (r^2=0.996) \qquad [S.1]$$

$$T1_{corrected} = -0.000134 \cdot {T1_{DESPOT}}^2 + 0.870 \cdot T1_{DESPOT} + 15.2 \; (r^2=0.997) \qquad [S.2]$$

The above 2nd order polynomial in [S.2] (r^2=0.997) was preserved for longer TRs with the 1st order coefficient increasing to 0.925 (r^2=0.998) and 1.02 (r^2=0.999) for TR=40 ms and TR=50 ms, respectively. Equation [S.2] was used to correct all *ex vivo* T1s as the RMSE for the 2nd order polynomial was always less than that of the first order polynomial.
