# Supplementary figures and images for: Native contrast visualization and tissue characterization of myocardial radiofrequency ablation and acetic acid chemoablation lesions at 0.55 T
Source: J Cardiovasc Magn Reson. 2021 May 6;23:50. doi: 10.1186/s12968-020-00693-1 (PMC8101152; doi:10.1186/s12968-020-00693-1)

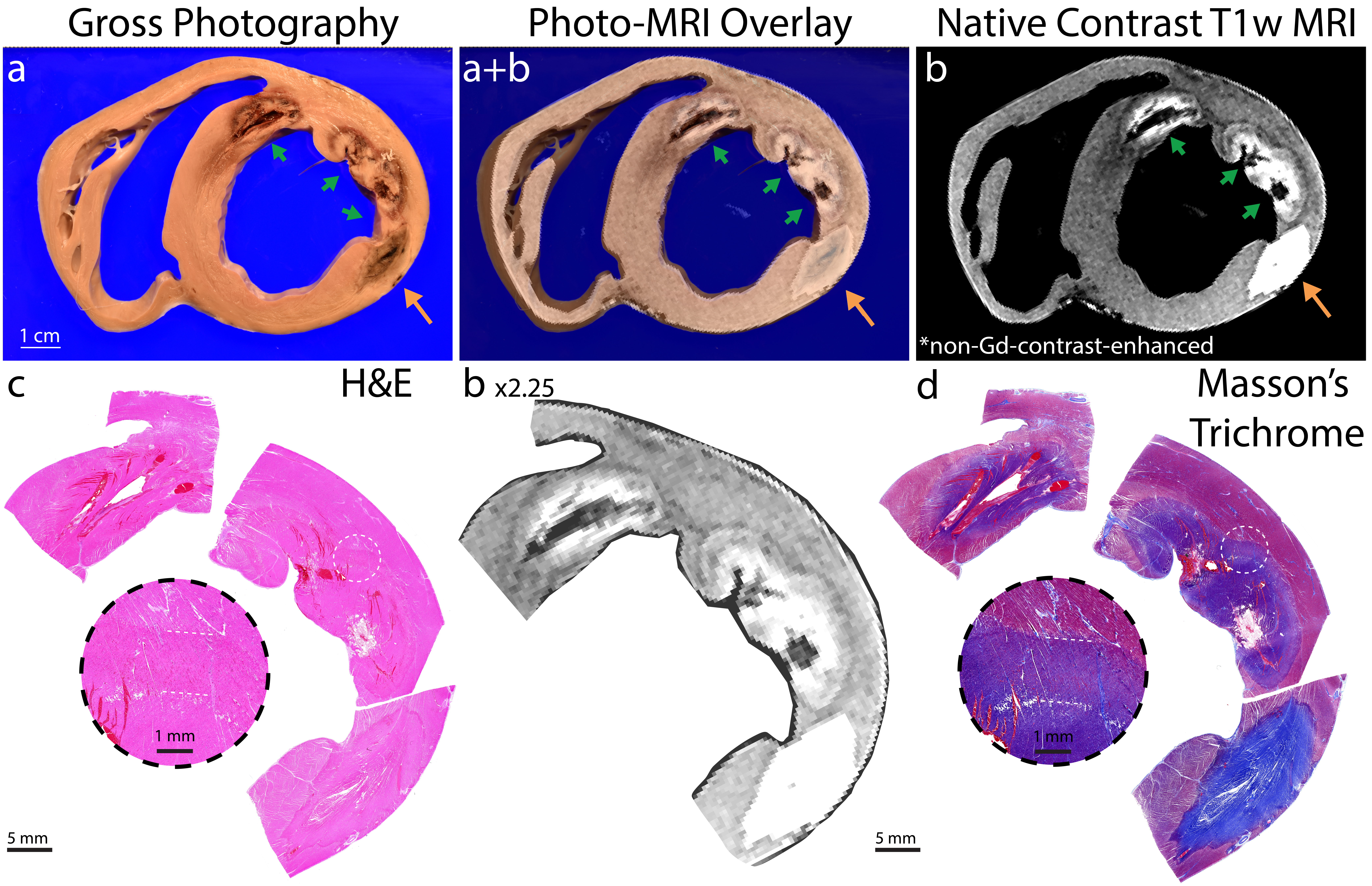

Supplement: Supplementary file 3 — Additional file 3: Figure S2.Visual correlation of RF ablation and chemoablation lesions acutely on Day 0 after ablation using gross photography, native contrast CMR and histological stains. Gross photography (a) compares very well with CMR (b) as shown by the overlay of the two (a + b). RF ablation (green arrows) and chemoablation (orange arrows) lesions are clearly seen including the boundary between T1w enhancing and non-enhancing portions of RF ablation lesions. This boundary can also be visualized on histological stains (c, d). Chemoablation lesions do not exhibit a significant non-T1w enhancing peripheral zone. The areas of necrosis can be visualized as purple in Masson’s Trichrome stain, while small pockets of blood can be most easily visualized in the H&E stain. The hypointense cavity within the septal RF ablation lesion is due to a “steam-pop” resulting from tissue overheating. Overall, there is excellent correlation between areas of T1w enhancement and the inner portion of the necrotic core of lesions. [file 12968_2020_693_MOESM3_ESM.png]

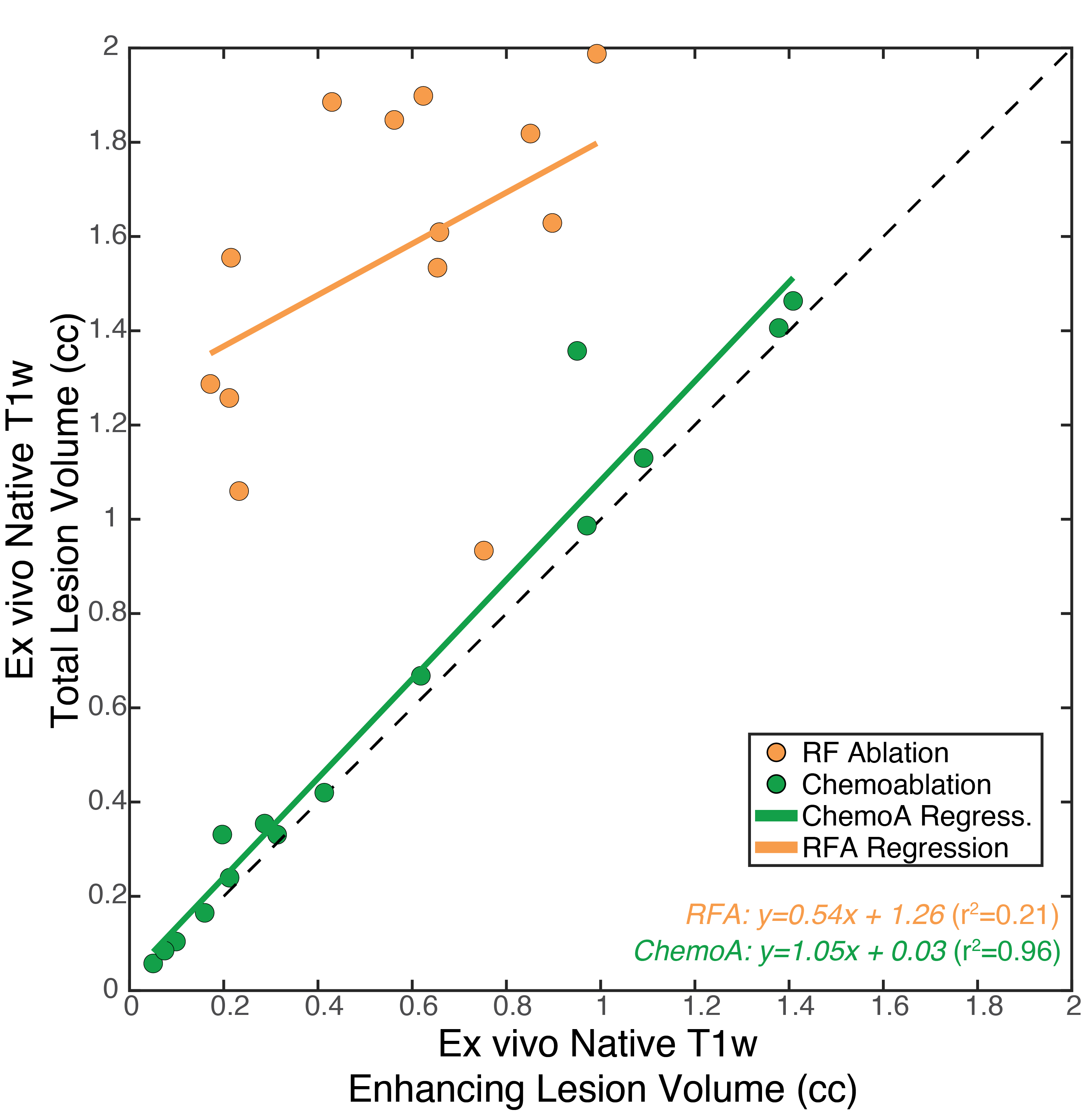

Supplement: Supplementary file 6 — Additional file 6: Figure S3.Comparing T1w enhancing volume and total lesions volume both from native contrast ex vivo imaging measures the fraction of each lesion that enhances. On average across all non-confluent lesions 89 ± 11% and 36 ± 19% of total lesion volume enhances for chemoablation and RF ablation, respectively. [file 12968_2020_693_MOESM6_ESM.png]
